# The Clinical Factors Associated with Clazosentan Discontinuation and Exploratory Associations with Discharge and Six-Month Outcomes in Aneurysmal Subarachnoid Hemorrhage: A Multicenter DCI Japan Registry Study

**DOI:** 10.3390/jcm15176744

**Published:** 2026-08-30

**Authors:** Yusuke Inoue, Masahito Katsuki, Toshikazu Hidaka, Yukishige Hashimoto, Yusuke Yamamoto, Daizo Ishii, Katsumi Takizawa, Yoji Tanaka, Masaki Chin, Motohiro Morioka, Hiroki Kurita, Takatoshi Sorimachi, Koreaki Irie, Ichiro Nakahara, Nobutaka Horie, Yasuhiko Matsumori, Fusao Ikawa

**Affiliations:** 1 Department of Neurosurgery, Shimane Prefectural Central Hospital, 4-1-1 Himebara, Izumo 693-8555, Shimane, Japan; yusukeinoue0610@outlook.jp (Y.I.);; 2 Physical Education and Health Center, Nagaoka University of Technology, Nagaoka 940-2137, Niigata, Japan; 3 Insight Research Ireland Centre, School of Health and Human Performance for Data Analytics, Dublin City University, D09 YT18 Dublin, Ireland; 4 Department of Neurosurgery, Graduate School of Biomedical and Health Sciences, Hiroshima University, Hiroshima 734-8551, Hiroshima, Japan; 5 Department of Neurosurgery, Japanese Red Cross Asahikawa Hospital, Asahikawa 070-0061, Hokkaido, Japan; 6 Department of Neurosurgery, Kyorin University, Mitaka 181-8611, Tokyo, Japan; 7 Department of Neurosurgery, Kurashiki Central Hospital, Kurashiki 710-0052, Okayama, Japan; 8 Department of Neurosurgery, Kurume University School of Medicine, Kurume 830-0011, Fukuoka, Japan; 9 Department of Cerebrovascular Surgery, Saitama Medical University International Medical Center, Hidaka 350-1298, Saitama, Japan; 10 Department of Neurosurgery, Tokai University, Isehara 259-1193, Kanagawa, Japan; 11 Department of Neurosurgery, Japan Red Cross Medical Center, Shibuya 150-8935, Tokyo, Japan; 12 Department of Neurosurgery, Fukuoka Shin Mizumaki Hospital, Mizumaki 807-0051, Fukuoka, Japan; 13 Sendai Headache and Neurology Clinic, Sendai 982-0014, Miyagi, Japan

**Keywords:** adverse events, cerebral vasospasm (VS), clazosentan, elderly, subarachnoid hemorrhage (SAH)

## Abstract

**Background/Objectives**: Subarachnoid hemorrhage (SAH) is a life-threatening condition frequently complicated by cerebral vasospasm (VS). Clazosentan has been shown to reduce the incidence of VS and improve outcomes in Japanese studies, and it has been available in Japan since January 2022. However, clazosentan has been associated with systemic adverse events, sometimes leading to discontinuation. This retrospective observational study investigated clinical variables associated with clazosentan discontinuation. **Methods**: We analyzed data from the multicenter “Database of Cohort Study for Outcome of SAH in Japan” (DCI Japan), prospectively collected from 2020 to 2024. Patients with aneurysmal SAH who underwent surgical clipping or endovascular coiling less than 4 days from onset were included. Multivariable logistic regression was used to investigate variables associated with clazosentan discontinuation. Secondary exploratory analyses examined clinical variables associated with poor functional outcomes (modified Rankin Scale [mRS] 3–6) at discharge and 6 months. **Results**: Among 372 patients treated with clazosentan (mean age 64.2 ± 14.3 years; 72.6% female), 91.7% (341/372) completed clazosentan during the VS phase, while 8.3% (31/372) discontinued clazosentan prematurely, mainly due to pulmonary complications. Overall, 8.9% (31/350) developed symptomatic VS, and 46.0% (171/372) had poor outcomes (modified Rankin Scale 3–6) at discharge and 31.4% (106/338) at 6 months. Clazosentan discontinuation was associated with age ≥ 76 years (OR for discontinuation: 2.33, 95% CI 1.03–5.28), World Federation of Neurosurgical Societies Grade V (OR 6.52 [2.70–15.78]), larger aneurysm size (OR 1.15 [1.03–1.29]), Fisher computed tomography group 4 (OR 2.61 [1.08–6.30]), and Fasudil hydrochloride hydrate use (OR 3.96 [1.77–8.87]). Poor functional outcome was more frequent in the discontinuation group than in the completion group both at discharge (83.9% vs. 42.5%, *p* < 0.001) and at 6 months (82.8% vs. 26.5%, *p* < 0.001). Clazosentan discontinuation was associated with poor outcome at discharge and at 6 months, although the latter was attenuated in sensitivity analysis. **Conclusions**: Some factors were associated with clazosentan discontinuation, highlighting the clinical relevance of careful systemic monitoring during clazosentan therapy, particularly in patient populations underrepresented in prior trials.

## 1. Introduction

Subarachnoid hemorrhage (SAH) is a severe neurological disorder associated with high morbidity and mortality [1,2]. Cerebral vasospasm (VS), a major complication of SAH, contributes to delayed cerebral ischemia (DCI) and poor clinical outcomes [3]. VS develops in 30–70% of SAH cases and results in stroke or death in 15–20% [4]. Although the pathogenesis of DCI is multifactorial [4], the prevention of VS remains an important therapeutic goal. Current pharmacologic strategies include nimodipine, a calcium channel blocker widely used in Western countries [5], and fasudil hydrochloride hydrate (Fasudil), a Rho kinase inhibitor commonly prescribed in Japan [6].

Clazosentan, a selective endothelin A receptor antagonist, inhibits endothelin-1–mediated vasoconstriction [7]. While the CONSCIOUS trials demonstrated its ability to reduce VS, they showed limited impact on functional outcome and mortality [8,9,10]. Similarly, the REACT trial [11] did not demonstrate a significant reduction in DCI. In contrast, a phase 3 randomized controlled trial (RCT) conducted in Japan [12] reported that clazosentan reduced VS and improved 12-week clinical outcomes. Since clazosentan became available for clinical use in Japan in January 2022 [13], these favorable findings have been further supported by several retrospective real-world studies [14,15,16,17,18,19,20,21,22,23,24,25,26,27,28,29].

However, despite its efficacy in vasospasm prevention, clazosentan has been associated with systemic adverse events, with an incidence of approximately 30%. Cardiopulmonary complications, such as pulmonary edema and hypotension, and other conditions pose significant challenges in clinical management, necessitating early clazosentan discontinuation in 5.8–11.7% of total patients treated with clazosentan [8,9,10,11,12]. Clazosentan discontinuation therefore represents a clinically meaningful and challenging event, often reflecting critical hemodynamic instability [30]. In addition, clazosentan discontinuation as a prophylactic therapy during the VS phase may compromise its protective effect against VS and probably identify patients with systemic vulnerability. Nevertheless, the incidence, risk factors, and clinical consequences of clazosentan discontinuation have not been fully characterized in a large real-world cohort.

Furthermore, in the Japanese phase 3 RCT, older patients (≥76 years), those with World Federation of Neurosurgical Societies (WFNS) Grade V, those belonging to Fisher computed tomography (CT) group 1–2 or 4, or those receiving combination prophylactic therapies during the VS phase were excluded [12]. In particular, Fasudil is sometimes co-administered with clazosentan for vasospasm prevention [31], but this combination was excluded from the Japanese phase 3 RCT, leaving its safety and discontinuation risk uncharacterized. Consequently, the safety and effectiveness of clazosentan in these conditions and populations remain insufficiently characterized.

Accordingly, this multicenter retrospective observational study primarily aimed to identify clinical factors associated with clazosentan discontinuation. As a secondary exploratory objective, we examined the associations between clazosentan discontinuation, symptomatic vasospasm, and functional outcome at discharge and 6 months. We also performed an exploratory subgroup analysis in patients aged ≥75 years, a clinically relevant elderly population in Japan.

## 2. Materials and Methods

### 2.1. Ethical Considerations

This multicenter study was approved by the institutional review boards of all participating centers; the representative ethical approval was obtained from the Shimane Prefectural Central Hospital Ethics Committee (Approval No. R22-020). Informed consent was waived under an opt-out policy. The study was conducted in accordance with the Declaration of Helsinki and the STROBE guidelines.

### 2.2. Dataset Information

This study used data from the “Database of Cohort Study for Outcome of SAH In Japan (DCI Japan),” a multicenter prospective registry of ten high-volume cerebrovascular centers in Japan [32]. Details of the study protocol of DCI Japan are provided in Supplementary File S1. Consecutive cases were enrolled between January 2020 and December 2024. The registry prospectively collected baseline characteristics, treatment details, VS-related variables, and functional outcomes, and we analyzed these data retrospectively.

### 2.3. Consistent Treatment Protocol of General Management for Aneurysmal SAH

General management of aSAH followed the Japanese Guidelines for the Management of Stroke 2021 [33] and local institutional protocols. Details are provided in Supplementary File S2.

### 2.4. Inclusion and Exclusion Criteria

**Figure 1:**
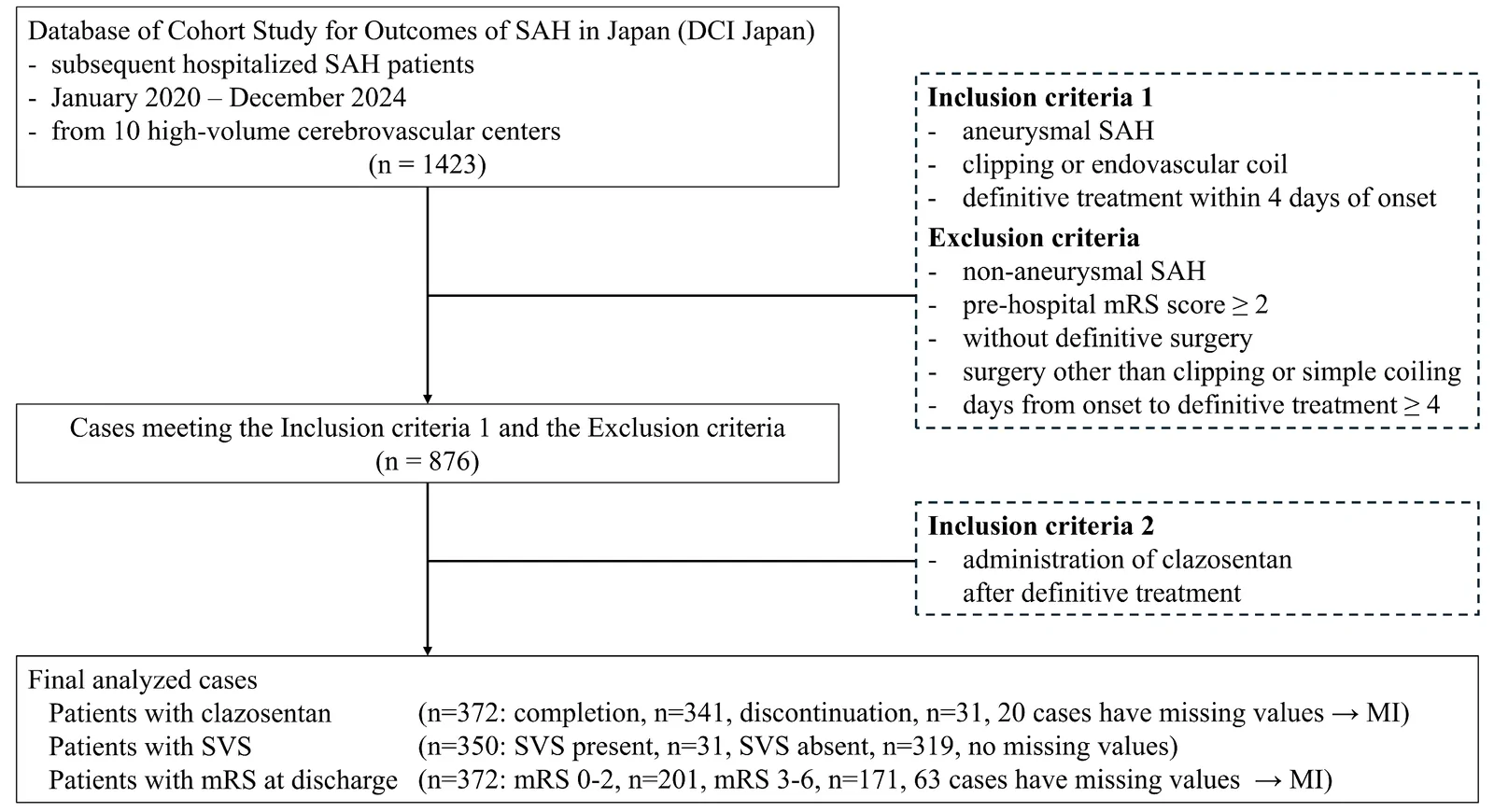
A flow diagram illustrating patient selection and exclusion criteria for the present study. Of the 1423 patients enrolled in the DCI Japan registry, 372 patients treated with clazosentan were included in the final analysis. Abbreviations: MI, multiple imputation; mRS, modified Rankin Scale; SAH, subarachnoid hemorrhage; SVS, symptomatic vasospasm.

A flow diagram of patient selection is shown in Figure 1. We included patients with aSAH who underwent surgical clipping or endovascular coiling for a ruptured aneurysm less than 4 days of symptom onset and subsequently received clazosentan. Patients with non-aneurysmal SAH, non-saccular aneurysms, poor premorbid status, no definitive aneurysm treatment, complex or crossover procedures, missing key dates, or treatment ≥ 4 days after onset were excluded. Detailed criteria are provided in Supplementary File S2.

### 2.5. Definition of SVS

SVS [34] was defined as a new focal neurological deficit or a ≥2-point decrease in Glasgow Coma Scale lasting at least 1 h during days 3–14 after aSAH onset, after excluding other causes of neurological worsening [35]. Detailed definitions of AVS and cerebral infarction [3,34,35,36,37,38] are provided in Supplementary File S2.

### 2.6. Management of VS

Before the approval of clazosentan in Japan (January 2022), VS prophylaxis was performed with intravenous Fasudil (90 mg/day for 14 days) [33]. Fasudil is considered one of the standard pharmacologic options for vasospasm prevention in Japan and has been widely used in routine clinical practice. After approval, clazosentan (10 mg/h for 14 days) was administered within 24 h of definitive aneurysm treatment as VS prophylaxis in eligible patients, and clazosentan was sometimes administered concomitantly with Fasudil or other prophylaxis in real-world practice [31]. Clazosentan was administered based on clinical availability and physician discretion [31], and was not used reactively for established VS.

Because clazosentan became available in Japan in January 2022, patients enrolled before its approval were not candidates for treatment. After approval, the decision to administer clazosentan was based on drug availability, institutional practice, and the treating physician’s clinical judgment. The registry did not collect standardized reasons for non-administration.

Clazosentan discontinuation was recorded as a study variable. In analyses of prophylactic drugs for VS, clazosentan was included regardless of whether the full 14-day administration was completed. Patients who received clazosentan continuously for 14 days were classified as the “completion group,” whereas those whose treatment was discontinued before day 14 were classified as the “discontinuation group.” Reasons for discontinuation were collected and categorized using a multiple-response format, including pulmonary complications, cardiac complications, hypotension, and other causes. The exact timing of clazosentan discontinuation during the treatment course was not available across centers and was therefore not incorporated into time-dependent analyses. Therefore, we could not distinguish early from late discontinuation or establish the temporal sequence between adverse events, treatment discontinuation, SVS, and other outcomes.

Additional therapies, including Fasudil, cilostazol, statins (rosuvastatin, atorvastatin, pitavastatin), and intravenous nicardipine, were administered as clinically indicated. The use of these prophylactic drugs during the VS phase was defined as treatment initiated the day after surgery and continued for 14 days.

### 2.7. Variables and Outcomes

Collected data included age, sex, history of hypertension, diabetes mellitus, stroke, WFNS Grade [39], aneurysm size and location, Fisher CT group [40], treatment modality (surgical clipping or endovascular coiling), periprocedural management, and VS prophylaxis during the VS phase, including spinal/ventricular/cisternal drains, clazosentan completion/discontinuation, concomitant use of Fasudil, cilostazol, statins, and nicardipine. Antiepileptic drug use (levetiracetam, lacosamide, perampanel) was also recorded. However, the timing of seizures relative to SAH onset was not available; therefore, early seizures could not be specifically identified. Radiological interventions for VS included intra-arterial Fasudil and angioplasty. Cerebral complications (hemorrhage, edema, infarction, infection) and systemic complications (infections requiring antibiotics, syndrome of inappropriate secretion of antidiuretic hormone, lung edema, arrhythmia) were documented.

The primary outcome was clazosentan discontinuation. Secondary exploratory outcomes were the observed occurrence of SVS and mRS (0–2 vs. 3–6) at discharge and 6 months. Functional status after aSAH may continue to improve after hospital discharge. This was because previous longitudinal observations have shown substantial recovery in mRS during the first 6 months after SAH [41]. An additional exploratory subgroup analysis was performed among patients aged ≥75 years. Secondary analyses were intended to be descriptive and hypothesis-generating rather than causal. These associations were evaluated using the multivariable analyses described below.

Cause-specific mortality and the vascular territory of cerebral infarction were not collected as standardized registry variables. Cognitive outcomes were also unavailable.

### 2.8. Statistical Analyses

Normality of continuous variables was assessed using the Shapiro–Wilk test. Continuous variables are presented as means (SD) or medians [Q1–Q3], and categorical variables as numbers and percentages. Group comparisons were performed using *t*-tests, Mann–Whitney U tests, chi-square tests, or Fisher’s exact tests, as appropriate. Variables significant in univariable analyses were entered into multivariable logistic regression models together with age ≥ 76 years, sex, WFNS Grade V, and Fisher CT group. These variables were included to evaluate the influence of underrepresented patient subgroups in the Japanese phase 3 RCT [12]. Firth’s penalized likelihood method was used because of the limited number of events. Missing covariate data were handled using five imputations for the clazosentan discontinuation, discharge outcome, and 6-month outcome models, and pooled results were reported as the main analyses. For the 6-month outcome model, multiple imputation of missing covariates was performed among patients with available 6-month mRS. Because no covariate data were missing in the symptomatic vasospasm model, that analysis used complete covariate data among patients with available SVS outcomes; complete-case analyses are shown in Supplementary Tables S1–S3. Odds ratios and 95% confidence intervals were calculated. Two-tailed *p* < 0.05 was considered statistically significant. Further details are provided in Supplementary File S2.

An additional exploratory subgroup analysis was performed among patients aged ≥75 years [42,43,44]. Age ≥ 76 years was used in the multivariable models to reflect the eligibility criteria of the Japanese phase 3 trial, whereas age ≥ 75 years was used for the exploratory elderly subgroup based on previous studies of elderly patients with aSAH [42,43,44] and the age threshold used in Japan’s healthcare system for older adults. Because of the limited number of clazosentan discontinuation events in this subgroup, the analysis was primarily descriptive and univariable, and no additional multivariable model was fitted.

## 3. Results

### 3.1. Patient Characteristics

Of the 1423 enrolled patients, 372 received clazosentan and were included in the analysis (Figure 1).

The mean age was 64.2 (14.3) years, and 72.6% were female. The median WFNS Grade was II (I–IV), and the median Fisher CT group was 3 (3–3). Endovascular coiling was performed in 47.0% (175/372) of cases. Among these patients, 91.7% (341/372) completed clazosentan during the VS phase, while 8.3% (31/372) discontinued clazosentan prematurely (Table 1). Reasons for discontinuation were pulmonary complications (29.0%, 9/31); cardiac complications (25.8%, 8/31); hypotension (12.9%, 4/31); cerebral edema (12.9%, 4/31); deterioration of general condition (9.7%, 3/31); intracranial hemorrhage (6.5%, 2/31); and septic shock (3.2%, 1/31).

Overall, 8.9% (31/350) of patients with available SVS data developed SVS, and poor outcomes (mRS 3–6) were observed in 46.0% (171/372) at discharge. The incidence of SVS and poor outcomes at discharge was higher among patients who discontinued clazosentan (Table 2).

### 3.2. Clinical Variables Associated with Clazosentan Discontinuation

Using the significant variables from the univariable analysis associated with clazosentan discontinuation (Table 1), we performed a multivariable analysis to identify factors associated with clazosentan discontinuation. The multivariable analysis with five imputations revealed that clazosentan discontinuation was associated with age ≥ 76 years (OR for discontinuation: 2.33, 95% CI 1.03–5.28); WFNS Grade V (OR 6.52 [2.70–15.78]); larger aneurysm size (OR 1.15 [1.03–1.29]); Fisher CT group 4 (OR 2.61 [1.08–6.30]); and Fasudil use (OR 3.96 [1.77–8.87]) (Table 3). The complete-case sensitivity analysis (*n* = 352) confirmed similar trends (Supplementary Table S1).

### 3.3. Secondary Exploratory Analysis: Clinical Variables Correlated with the Observed Occurrence of SVS

Using the significant variables from the univariable analysis, along with age, sex, WFNS Grade, and Fisher CT group we performed a multivariable analysis. Secondary exploratory analyses showed that WFNS Grade V (OR 2.57 [1.06–6.21]) and clazosentan discontinuation (OR 2.84 [1.01–8.03]) were correlated with the observed occurrence of SVS among 350 patients with available SVS data (Table 4).

### 3.4. Secondary Exploratory Analysis: Clinical Variables Correlated with the Observed Poor Outcomes (mRS 3–6) at Discharge

The multivariable analysis with five imputations revealed that age ≥ 76 years (OR 8.03 [4.01–16.10]), WFNS Grade V (OR 7.37 [1.55–35.01]), Fisher CT group 4 (OR 9.98 [3.40–29.30]), ventricular drainage (OR 2.15 [1.19–3.87]), statin use (OR 3.06 [1.63–5.76]), clazosentan discontinuation (OR 5.08 [2.36–10.90]), the presence of SVS (OR 3.31 [1.15–9.55]), and systemic complication (OR 5.00 [2.26–11.10]) were correlated with poor outcomes (Table 5). The complete-case sensitivity analysis (*n* = 309) confirmed similar trends (Supplementary Table S2).

### 3.5. Secondary Exploratory Analysis: Clinical Variables Correlated with the Observed Poor Outcomes (mRS 3–6) at 6 Months

Among the entire cohort, 6-month mRS was available in 338 patients, of whom 106 (31.4%) had a poor functional outcome (mRS 3–6). A poor 6-month outcome was observed in 82/309 (26.5%) patients in the completion group and 24/29 (82.8%) patients in the discontinuation group (*p* < 0.001). The multivariable analysis with five imputations revealed that age ≥ 76 years (OR 4.21 [1.95–9.10]), WFNS Grade V (OR 5.27 [2.48–11.19]), Fisher CT group 4 (OR 12.06 [4.18–34.83]), ventricular drainage (OR 2.27 [1.04–4.98]), statin use (OR 3.24 [1.48–7.08]), clazosentan discontinuation (OR 5.12 [1.41–18.60]), presence of SVS (OR 10.58 [3.28–34.16]), and systemic complication (OR 7.62 [3.28–17.74]) were correlated with poor outcomes at 6 months (Table 6). However, in the complete-case sensitivity analysis (*n* = 278), the association between clazosentan discontinuation and poor 6-month outcome was attenuated and did not reach statistical significance (OR 3.83 [0.90–16.33], *p* = 0.070) (Supplementary Table S3).

### 3.6. Exploratory Analyses of Patients Aged ≥75 Years and Six-Month Functional Outcomes

Among 111 patients aged ≥75 years, 91 (82.0%) completed clazosentan treatment and 20 (18.0%) discontinued treatment. The discontinuation group had higher WFNS Grades (*p* = 0.006) and a different distribution of Fisher CT groups (*p* = 0.006) than the completion group (Supplementary Table S4). A poor functional outcome (mRS 3–6) was more frequent in the discontinuation group both at discharge (100.0% vs. 68.1%, *p* = 0.008) and at 6 months among patients with available follow-up (88.9% vs. 45.3%, *p* = 0.002). SVS was also more frequent in the discontinuation group (29.4% vs. 6.8%, *p* = 0.016), as were cerebral complications (55.0% vs. 26.4%, *p* = 0.026) and systemic complications (55.0% vs. 27.5%, *p* = 0.034) (Supplementary Table S5). Given the limited number of discontinuation events (*n* = 20), no multivariable analysis was performed for this exploratory subgroup.

## 4. Discussion

In this multicenter retrospective observational study of 372 patients with aSAH, clazosentan discontinuation was associated with age ≥ 76 years, WFNS Grade V, Fisher CT group 4, larger aneurysm size, and concomitant Fasudil use. These factors represent patient populations with heightened systemic vulnerability. Secondary exploratory analyses showed associations between discontinuation and SVS or poor discharge outcome, but these findings likely reflect underlying disease severity and systemic instability rather than a causal effect. Clazosentan discontinuation may therefore be regarded as a clinical marker of severe neurological or systemic deterioration in real-world practice.

### 4.1. Comparison with RCTs and Previous Observational Studies

In prior RCTs of clazosentan (Table 7) [8,9,10,11,12], treatment discontinuation due to adverse events occurred in approximately 5.8–11.7% of patients, with pulmonary and hemodynamic complications consistently recognized as major safety concerns [8,9,10,11,12]. In the present real-world cohort, the discontinuation rate was 8.3%, which was comparable to these RCTs, and cardiopulmonary complications accounted for most discontinuations. Although the study designs and patient populations differed between the trials and our cohort, the overall frequency and pattern of discontinuation were largely consistent. These findings suggest that clazosentan discontinuation due to systemic adverse events is not an isolated event, but a reproducible clinical issue across different treatment settings.

For contextual comparison, we reviewed real-world observational studies published after PMDA approval of clazosentan in January 2022. The reported incidence of non-ischemic adverse events, including pulmonary edema and pleural effusion, ranged from 19.8% to 56.3%, and discontinuation rates ranged from 0% to 50% (Table 8) [14,15,16,17,18,19,20,21,22,23,24,25,26,27,28,29]. This wide range likely reflects differences in patient selection, treatment protocols, disease severity, and systemic management.

Nevertheless, cardiopulmonary and fluid-related complications were consistently observed as major reasons for discontinuation. Therefore, clazosentan discontinuation should be regarded not simply as failure of prophylactic therapy, but as a clinical signal of evolving systemic stress. More standardized fluid management, baseline laboratory assessment, and cardiopulmonary monitoring may help improve treatment completion, particularly in patients at high risk of discontinuation [17,22,28].

### 4.2. Factors Associated with Clazosentan Discontinuation

In this study, clazosentan discontinuation was associated with advanced age (≥76 years), WFNS Grade V, Fisher CT group 4, and concomitant Fasudil use. These factors likely identify patients with limited physiological reserve and greater vulnerability to systemic complications during the acute phase of SAH, rather than representing independent causes of poor outcome. Although disease severity may partly confound these associations, the pharmacological profile of clazosentan and the systemic instability of severe SAH provide plausible explanations for difficulty completing treatment.

Advanced age may increase susceptibility to clazosentan-related adverse events. Clazosentan is predominantly eliminated via biliary excretion mediated by organic anion-transporting polypeptides 1B1 and 1B3 [45]. Age-related changes in hepatic function, transporter activity, and cardiopulmonary or renal reserve may reduce tolerance to hemodynamic stress and fluid retention [27].

Similarly, WFNS Grade V and Fisher CT group 4 reflect severe initial hemorrhage and are associated with systemic complications, including pulmonary dysfunction, cardiac instability, infection, and disturbances of sodium and water balance [46]. Intracerebral or intraventricular hemorrhage, which characterizes Fisher CT group 4, may further increase treatment intensity, systemic inflammation, and difficulty in maintaining fluid balance [47].

Concomitant Fasudil use was also associated with clazosentan discontinuation. Although Fasudil alone has not been reported to cause fluid retention, previous data suggest that combined Fasudil and clazosentan therapy may be associated with fluid retention [15]. Clazosentan induces vasodilation through endothelin-A receptor antagonism, whereas Fasudil promotes vasodilation via Rho-kinase inhibition. Their combined use may therefore increase vasodilatory burden and contribute to edema or hemodynamic instability, although this mechanism remains speculative. Moreover, prior studies have not clearly demonstrated an additive benefit of Fasudil for VS prevention [20]. These findings suggest that concomitant clazosentan and Fasudil therapy should be used cautiously, particularly in patients with high systemic vulnerability.

In the present study, clazosentan discontinuation showed a stronger association with poor outcomes at discharge than SVS did; however, this should not be interpreted as causal. Rather, discontinuation may capture broader systemic deterioration that is closely linked to early neurological outcomes. From a clinical perspective, careful patient selection and proactive management of cardiopulmonary stability and fluid balance may be important for optimizing safe clazosentan therapy. Future prospective studies with standardized protocols and time-resolved exposure data are needed to determine whether specific management strategies can reduce discontinuation while maintaining efficacy.

### 4.3. Subgroup Analysis Among Patients Aged ≥75

In the exploratory subgroup of patients aged ≥75 years, clazosentan discontinuation occurred in 18.0% and was accompanied by more severe neurological presentation and markedly poorer functional outcomes. These findings support careful cardiopulmonary and systemic monitoring when clazosentan is administered to older patients, a population that was underrepresented in the pivotal Japanese phase 3 trial. However, because only 20 discontinuation events occurred in this subgroup, adjusted effect estimates would be unstable, and the findings should be considered descriptive and hypothesis-generating.

### 4.4. Limitations

First, the retrospective observational design, modest sample size, and missing data preclude causal inference. Clazosentan discontinuation was often triggered by systemic complications and should be interpreted as a marker of disease severity and systemic vulnerability rather than a direct cause of SVS or poor outcomes. The functional outcome model also included post-treatment variables, which may have introduced path-specific confounding. Second, data on baseline cardiopulmonary and renal function, fluid balance, diuretic use, albumin supplementation, and the timing of discontinuation were unavailable. Also, hydrocephalus was not captured as a separate standardized variable in the registry. Ventricular drainage was available but is an imperfect surrogate because it reflects both hydrocephalus and local treatment practice. Third, no registry-wide criteria were established for clazosentan initiation, interruption, or discontinuation. Fluid management, cardiopulmonary monitoring, concomitant prophylaxis, and decisions to discontinue treatment may have varied among institutions and physicians. In addition, clinical experience with clazosentan may have evolved between 2022 and 2024. Such institutional and temporal heterogeneity may have affected the observed discontinuation rate and associated factors. Fourth, the registry did not capture the timing of seizure occurrence, cause-specific mortality, vascular territory or volume of cerebral infarction [48], or standardized cognitive outcomes. Therefore, we could not evaluate the prognostic relevance of early seizures [49], distinguish neurological from non-neurological causes of death, examine territory-specific effects of DCI-related infarction, or assess long-term cognitive recovery. These variables should be prospectively collected in future studies. Fifth, multiple exploratory analyses were performed, and the limited number of discontinuation events may have reduced the precision of the estimates despite Firth’s penalized likelihood method. Sixth, SVS does not fully capture the multifactorial nature of DCI. These findings should therefore be considered hypothesis-generating and require validation in prospective studies with standardized systemic management and time-dependent exposure data.

Future prospective studies should incorporate standardized criteria for clazosentan initiation and discontinuation, time-resolved drug exposure, fluid balance and cardiopulmonary monitoring, cause-specific adverse events and mortality, seizure timing, and detailed imaging characterization of DCI-related infarction. Longer-term follow-up incorporating both functional and cognitive outcomes will also be important to determine whether strategies that improve treatment completion translate into meaningful patient-centered benefit.

## 5. Conclusions

Clazosentan discontinuation was associated with age ≥ 76 years, WFNS Grade V, Fisher CT group 4, larger aneurysm size, and concomitant Fasudil use. Its associations with SVS and poor outcomes at discharge and at 6 months likely reflect underlying disease severity and systemic vulnerability rather than a causal effect of discontinuation. Careful systemic monitoring may be particularly important when using clazosentan in patient populations underrepresented in prior RCTs. Prospective studies incorporating standardized treatment protocols, time-resolved exposure and adverse event data, and longer-term functional and cognitive outcomes are needed to determine how clazosentan can be used most safely and effectively in real-world aSAH care.

## Figures and Tables

**Table 1:** Clinical characteristics of patients with aneurysmal SAH treated with clazosentan.

	Overall (*n* = 372)	Completion Group (*n* = 341, 91.7%)	Discontinuation Group (*n* = 31, 8.3%)	*p* Value
Age (years), mean (SD)	64.2 (14.3)	63.2 (14.4)	76.0 (6.9)	<0.001 *
Age ≥ 76 years (%)	100 (26.9%)	84 (24.6%)	16 (51.6%)	<0.001 *
Female sex (%)	270 (72.6%)	249 (73.0%)	21 (67.7%)	0.674
Past history				
Hypertension (%)	131/344 (38.1%)	118/318 (37.1%)	13/26 (50.0%)	0.275
Diabetes mellitus (%)	33/347 (9.5%)	29/320 (9.1%)	4/27 (14.8%)	0.524
Stroke (%)	14/347 (4.0%)	14/320 (4.4%)	0/27 (0.0%)	0.548
WFNS Grade, median (IQR)	II (I–IV)	II (I–IV)	IV (IV–V)	<0.001 *^†^
I (%)	116 (31.2%)	114 (33.4%)	2 (6.5%)	
II (%)	95 (25.5%)	94 (27.6%)	1 (3.2%)	
III (%)	19 (5.1%)	17 (5.0%)	2 (6.5%)	
IV (%)	57 (15.3%)	46 (13.5%)	11 (35.5%)	
V (%)	85 (22.8%)	70 (20.5%)	15 (48.4%)	<0.001 *^‡^
Aneurysm size (mm), mean (SD)	5.9 (3.0) (*n* = 352)	5.7 (2.8) (*n* = 323)	7.8 (4.4) (*n* = 29)	0.007 *
Aneurysm location				
ACA or ACoA (%)	138 (37.1%)	130 (38.1%)	8 (25.8%)	
ICA (%)	121 (32.5%)	111 (32.6%)	10 (32.2%)	
MCA (%)	82 (22.0%)	72 (21.1%)	10 (32.3%)	
VA, BA, PCA, PICA (%)	30 (8.1%)	27 (7.9%)	3 (9.7%)	0.999 ^¶^
Fisher CT group, median (IQR)	3 (3–3)	3 (3–3)	3 (3–4)	0.002 *^†^
1 (%)	1 (0.3%)	1 (0.3%)	0 (0.0%)	
2 (%)	40 (10.8%)	38 (11.1%)	2 (6.5%)	
3 (%)	281 (75.5%)	263 (77.2%)	18 (58.0%)	
4 (%)	50 (13.4%)	39 (11.4%)	11 (35.5%)	0.003 *^§^
Surgical procedure				
Endovascular coiling, not surgical clipping (%endovascular coiling)	175 (47.0%)	161 (47.2%)	14 (45.2%)	0.975
Spinal drainage (%)	159 (42.7%)	144 (42.2%)	15 (48.4%)	0.636
Ventricular drainage (%)	121 (32.5%)	111 (32.6%)	10 (32.3%)	1.000
Cisternal drainage (%)	74 (19.9%)	72 (21.1%)	2 (6.5%)	0.085
Cerebral vasospasm prophylaxis and other medications				
Fasudil (%)	117 (31.5%)	100 (29.3%)	17 (54.8%)	0.003 *
Cilostazol (%)	262 (70.4%)	241 (70.7%)	21 (67.7%)	0.891
Statin (%)	159 (42.7%)	147 (43.1%)	12 (38.7%)	0.776
Nicardipine (%)	9 (2.4%)	3 (0.9%)	6 (19.4%)	0.006 *
Antiepileptic drug (%)	144 (38.7%)	134 (39.3%)	10 (32.3%)	0.563

Abbreviations: ACA, anterior cerebral artery; ACoA, anterior communicating artery; BA, basilar artery; CT, computed tomography; Fasudil, fasudil hydrochloride hydrate; ICA, internal carotid artery; IQR, interquartile range; MCA, middle cerebral artery; PCA, posterior cerebral artery; PICA, posterior inferior cerebellar artery; SAH, subarachnoid hemorrhage; SD, standard deviation; VA, vertebral artery; WFNS, World Federation of Neurosurgical Societies; *, *p* < 0.05. ^†^, treated as an ordinal variable, and tested by the Mann–Whitney U test. ^‡^, treated as a binomial variable, I–IV or V, and tested by the chi-square test. ^§^, treated as a three-categorical variable:—1 and 2, 3, or 4—and tested by the chi-square test. ^¶^, treated as a binomial variable, anterior circulation or posterior circulation, and tested by the chi-square test.

**Table 2:** Treatment outcomes of patients with SAH treated with clazosentan.

	Overall (*n* = 372)	Completion Group (*n* = 341, 91.7%)	Discontinuation Group (*n* = 31, 8.3%)	*p* Value
mRS score at discharge				<0.001 *^†^
0 (%)	82 (22.0%)	81 (23.8%)	1 (3.2%)	
1 (%)	66 (17.7%)	63 (18.5%)	3 (9.7%)	
2 (%)	53 (14.2%)	52 (15.2%)	1 (3.2%)	
3 (%)	39 (10.5%)	36 (10.6%)	3 (9.7%)	
4 (%)	43 (11.6%)	38 (11.1%)	5 (16.1%)	
5 (%)	67 (18.0%)	56 (16.4%)	11 (35.5%)	
6 (%)	22 (5.9%)	15 (4.4%)	7 (22.6%)	
Poor outcome at discharge (mRS 3–6) (%)	171/372 (46.0%)	145/341 (42.5%)	26/31 (83.9%)	<0.001 *
mRS score at 6 months (*n* = 338)				<0.001 *^†^
0 (%)	146/338 (43.2%)	145/309 (46.9%)	1/29 (3.4%)	
1 (%)	57/338 (16.9%)	54/309 (17.5%)	3/29 (10.3%)	
2 (%)	29/338 (8.6%)	28/309 (9.1%)	1/29 (3.4%)	
3 (%)	17/338 (5.0%)	15/309 (4.9%)	2/29 (6.9%)	
4 (%)	18/338 (5.3%)	14/309 (4.5%)	4/29 (13.8%)	
5 (%)	47/338 (13.9%)	36/309 (11.7%)	11/29 (37.9%)	
6 (%)	24/338 (7.1%)	17/309 (5.5%)	7/29 (24.1%)	
Poor outcome at 6 months (mRS 3–6) (%)	106/338 (31.4%)	82/309 (26.5%)	24/29 (82.8%)	<0.001 *
Complications				
Angiographic vasospasm (%)	66/354 (18.6%)	58/329 (17.6%)	8/25 (32.0%)	0.130
Symptomatic vasospasm (%)	31/350 (8.9%)	24/324 (7.4%)	7/26 (26.9%)	0.003 *
IVR against cerebral vasospasm (%)	11 (3.0%)	10 (2.9%)	1 (3.2%)	0.999
Cerebral infarction (%)	69/354 (19.5%)	58/327 (17.7%)	11/27 (40.7%)	0.008 *
Cerebral complication (%)	111 (29.8%)	92 (27.0%)	19 (61.3%)	<0.001 *
Systemic complication (%)	91 (24.5%)	75 (22.0%)	16 (51.6%)	0.001 *

Abbreviations: IVR, interventional radiology; mRS, modified Rankin Scale; SAH, subarachnoid hemorrhage. *, *p* < 0.05. ^†^, treated as an ordinal variable, and tested by the Mann–Whitney U test.

**Table 3:** Multivariable Firth’s penalized logistic regression model identifying clinical variables associated with clazosentan discontinuation with multiple imputations.

Variable	Odds Ratio (95% CI)	*p* Value
Age ≥ 76 years	2.33 (1.03–5.28)	0.042 *
Female sex	1.18 (0.54–2.56)	0.681
WFNS Grade V (vs. I–IV)	6.52 (2.70–15.78)	<0.001 *
Aneurysm size (mm)	1.15 (1.03–1.29)	0.021 *
Fisher CT group		
1–2	0.44 (0.14–1.34)	0.149
3	1.00 (Reference)	
4	2.61 (1.08–6.30)	0.033 *
Fasudil use	3.96 (1.77–8.87)	<0.001 *
Nicardipine use	1.22 (0.52–2.76)	0.742

Five imputed datasets were generated, and pooled estimates are presented. Variables that were significant in the univariable analysis (Table 1) were included in the multivariable model, along with age, sex, WFNS Grade, and Fisher CT group. Abbreviations: CI, confidence interval; CT, computed tomography; Fasudil, fasudil hydrochloride hydrate; WFNS, World Federation of Neurosurgical Societies; *, *p* < 0.05.

**Table 4:** Secondary exploratory multivariable Firth’s penalized logistic regression model examining clinical variables correlated with observed occurrence of symptomatic vasospasm (*n* = 350).

Variables	Univariable Analysis			Multivariable Analysis	
	Symptomatic Vasospasm (−) (*n* = 319, 91.1%)	Symptomatic Vasospasm (+) (*n* = 31, 8.9%)	*p* Value	OR for Symptomatic Vasospasm (+) (95% CI)	*p* Value
Age ≥ 76 years (%)	87 (27.3%)	9 (29.0%)	1.000	0.88 (0.38–2.05)	0.771
Female sex (%)	234 (73.4%)	24 (77.4%)	0.782	1.29 (0.56–2.97)	0.552
Past history					
Hypertension (%) (*n* = 324)	111/293 (37.9%)	8/31 (25.8%)	0.258		
Diabetes mellitus (%) (*n* = 326)	31/295 (10.5%)	0/31 (0.0%)	0.115		
Stroke (%) (*n* = 326)	10/295 (3.4%)	1/31 (3.2%)	1.000		
WFNS Grade, median (IQR)	II (I–IV)	IV (II–V)	<0.001 *^†^		
I (%)	107 (33.5%)	4 (12.9%)			
II (%)	85 (26.6%)	5 (16.1%)			
III (%)	16 (5.0%)	3 (9.7%)			
IV (%)	44 (13.8%)	8 (25.8%)			
V (%)	67 (21.0%)	11 (35.5%)	0.104 ^‡^	2.57 (1.06–6.21)	0.037 *
Aneurysm size (mm), mean (SD) (*n* = 333)	5.8 (2.8) (*n* = 304)	6.8 (4.8) (*n* = 29)	0.502		
Posterior circulation (VA, BA, PCA, PICA) (%)	27 (8.5%)	1 (3.2%)	0.497		
Fisher CT group, median (IQR)	3.0 (3.0–3.0)	3.0 (3.0–3.0)	0.245 ^†^		
1–2 (%)	32 (10.0%)	2 (6.5%)		0.74 (0.20–2.74)	0.650
3 (%)	248 (77.7%)	23 (74.2%)		1.00 (Reference)	
4 (%)	39 (12.2%)	6 (19.4%)	0.395 ^§^	1.29 (0.47–3.56)	0.626
Surgical procedure					
Endovascular coiling, not surgical clipping (%endovascular coiling)	154 (48.3%)	12 (38.7%)	0.407		
Spinal drainage (%)	144 (45.1%)	10 (32.3%)	0.234		
Ventricular drainage (%)	102 (32.0%)	14 (45.2%)	0.197		
Cisternal drainage (%)	70 (21.9%)	4 (12.9%)	0.344		
Cerebral vasospasm prophylaxis and other medications					
Fasudil (%)	99 (31.0%)	12 (38.7%)	0.500		
Cilostazol (%)	225 (70.5%)	23 (74.2%)	0.825		
Statin (%)	142 (44.5%)	14 (45.2%)	1.000		
Nicardipine (%)	6 (1.9%)	3 (9.7%)	0.043 *	3.21 (0.68–15.12)	0.140
Antiepileptic drug (%)	128 (40.1%)	16 (51.6%)	0.294		
Clazosentan discontinuation	19 (6.0%)	7 (22.6%)	0.003 *	2.84 (1.01–8.03)	0.048 *
Outcomes and complications					
mRS 3–6 at discharge (%)	133 (41.7%)	23 (74.2%)	0.001 *		
mRS 3–6 at 6 months (%) (*n* = 319)	76/294 (25.9%)	18/25 (72.0%)	<0.001 *		
Angiographic vasospasm (%) (*n* = 349)	32/319 (10.0%)	29/30 (96.7%)	<0.001 *		
IVR against cerebral vasospasm (%)	0 (0.0%)	7 (22.6%)	<0.001 *		
Cerebral infarction (%)	36 (11.3%)	29 (93.5%)	<0.001 *		
Cerebral complication (%)	72 (22.6%)	17 (54.8%)	<0.001 *		
Systemic complication (%)	61 (19.1%)	8 (25.8%)	0.511		

No missing data were present among covariates included in the multivariable model; therefore, the symptomatic vasospasm model used complete-case covariate data (outcome available-case, *n* = 350). We included the significant items from the univariable analysis, along with age, sex, WFNS Grade, and Fisher CT group. These analyses represent non-causal associations. Given the lack of time-resolved data on treatment discontinuation, the results should be interpreted as descriptive and exploratory. Abbreviations: BA, basilar artery; CI, confidence interval; CT, computed tomography; Fasudil, fasudil hydrochloride hydrate; IVR, interventional radiology; IQR, interquartile range; mRS, modified Rankin Scale; OR, odds ratio; PCA, posterior cerebral artery; PICA, posterior inferior cerebellar artery; SD, standard deviation; VA, vertebral artery; WFNS, World Federation of Neurosurgical Societies; *, *p* < 0.05. ^†^, treated as an ordinal variable, and tested by the Mann–Whitney U test. ^‡^, treated as a binomial variable, I–IV or V, and tested by the chi-square test. ^§^, treated as a three-categorical variable—1 and 2, 3, or 4—and tested by the chi-square test.

**Table 5:** Secondary exploratory multivariable Firth’s penalized logistic regression model examining clinical variables correlated with poor discharge outcomes (mRS 3–6) with multiple imputations.

Variables	Univariable Analysis			Multivariable Analysis	
	mRS 0–2 at Discharge (*n* = 201, 54.0%)	mRS 3–6 at Discharge (*n* = 171, 46.0%)	*p* Value	OR for mRS 3–6 (95% CI)	*p* Value
Age ≥ 76 years (%)	23 (11.4%)	77 (45.0%)	<0.001 *	8.03 (4.01–16.10)	<0.001 *
Female sex (%)	149 (74.1%)	121 (70.8%)	0.542	0.94 (0.47–1.89)	0.850
Past history					
Hypertension (%) (*n* = 345)	64/187 (34.2%)	67/158 (42.4%)	0.147		
Diabetes mellitus (%) (*n* = 347)	16/188 (8.5%)	17/159 (10.7%)	0.613		
Stroke (%) (*n* = 347)	3/188 (1.6%)	11/159 (6.9%)	0.025 *	3.35 (0.78–14.40)	0.101
WFNS Grade, median (IQR)	II (I–II)	IV (II–V)	<0.001 *^†^		
I (%)	94 (46.8%)	22 (12.9%)			
II (%)	64 (31.8%)	31 (18.1%)			
III (%)	10 (5.0%)	9 (5.3%)			
IV (%)	16 (8.0%)	41 (24.0%)			
V (%)	17 (8.5%)	68 (39.8%)	<0.001 *^‡^	7.37 (1.55–35.01)	0.013 *
Aneurysm size (mm), mean (SD) (*n* = 352)	5.3 (2.6)	6.5 (3.3)	0.021 *	1.08 (0.97–1.20)	0.171
Posterior circulation (VA, BA, PCA, PICA) (%)	15 (7.5%)	15 (8.8%)	0.786		
Fisher CT group	3.0 (3.0–3.0)	3.0 (3.0–3.0)	0.154 ^†^		
1–2 (%)	31 (15.4%)	10 (5.8%)		0.52 (0.20–1.35)	0.175
3 (%)	161 (80.1%)	118 (69.0%)		1.00 (Reference)	
4 (%)	9 (4.5%)	41 (24.0%)	<0.001 *^§^	9.98 (3.40–29.30)	<0.001 *
Surgical procedure					
Endovascular coiling, not surgical clipping (%endovascular coiling)	104 (51.7%)	71 (41.5%)	0.062		
Spinal drainage (%)	85 (42.3%)	74 (43.3%)	0.931		
Ventricular drainage (%)	48 (23.9%)	73 (42.7%)	<0.001 *	2.15 (1.19–3.87)	0.011 *
Cisternal drainage (%)	32 (15.9%)	42 (24.6%)	0.051		
Cerebral vasospasm prophylaxis and other medications					
Fasudil (%)	61 (30.3%)	56 (32.7%)	0.700		
Cilostazol (%)	144 (71.6%)	118 (69.0%)	0.659		
Statin (%)	75 (37.3%)	84 (49.1%)	0.029 *	3.06 (1.63–5.76)	<0.001 *
Nicardipine (%)	3 (1.5%)	6 (3.5%)	0.356		
Antiepileptic drug (%)	79 (39.3%)	65 (38.0%)	0.882		
Clazosentan discontinuation (%)	2 (1.0%)	29 (17.0%)	<0.001 *	5.08 (2.36–10.90)	<0.001 *
Outcomes and complications					
mRS 3–6 at 6 months (%) (*n* = 338)	2/193 (1.0%)	104/145 (71.7%)	<0.001 *		
Angiographic vasospasm (%) (*n* = 354)	24/194 (12.4%)	42/160 (26.2%)	0.001 *		
Symptomatic vasospasm (%) (*n* = 350)	8/194 (4.1%)	23/156 (14.7%)	0.001 *	3.31 (1.15–9.55)	0.026 *
IVR against cerebral vasospasm (%)	5 (2.5%)	6 (3.5%)	0.760		
Cerebral infarction (%) (*n* = 354)	22/194 (11.3%)	47/160 (29.4%)	<0.001 *		
Cerebral complication (%)	34 (16.9%)	77 (45.0%)	<0.001 *		
Systemic complication (%)	31 (15.4%)	60 (35.1%)	<0.001 *	5.00 (2.26–11.10)	<0.001 *

Five imputed datasets were generated, and pooled estimates are presented. Variables significant in the univariable analysis were included in the multivariable model, along with age, sex, WFNS Grade, and Fisher CT group. These analyses represent non-causal associations. Given the lack of time-resolved data on treatment discontinuation, the results should be interpreted as descriptive and exploratory. Abbreviations: BA, basilar artery; CI, confidence interval; CT, computed tomography; Fasudil, fasudil hydrochloride hydrate; IVR, interventional radiology; IQR, interquartile range; mRS, modified Rankin Scale; OR, odds ratio; PCA, posterior cerebral artery; PICA, posterior inferior cerebellar artery; SD, standard deviation; VA, vertebral artery; WFNS, World Federation of Neurosurgical Societies; *, *p* < 0.05. ^†^, treated as an ordinal variable, and tested by the Mann–Whitney U test. ^‡^, treated as a binomial variable, I–IV or V, and tested by the chi-square test. ^§^, treated as a three-categorical variable—1 and 2, 3, or 4—and tested by the chi-square test.

**Table 6:** Secondary exploratory multivariable Firth’s penalized logistic regression model examining clinical variables correlated with poor outcomes (mRS 3–6) at 6 months with multiple imputations.

Variables	Univariable Analysis			Multivariable Analysis	
	mRS 0–2 at 6 Months (*n* = 232, 68.6%)	mRS 3–6 at 6 Months (*n* = 106, 31.4%)	*p* Value	OR for mRS 3–6 (95% CI)	*p* Value
Age ≥ 76 years (%)	37 (15.9%)	45 (42.5%)	<0.001 *	4.21 (1.95–9.10)	<0.001 *
Female sex (%)	172 (74.1%)	73 (68.9%)	0.381	0.85 (0.42–1.71)	0.643
Past history					
Hypertension (%) (*n* = 310)	71/212 (33.5%)	43/98 (43.9%)	0.102		
Diabetes mellitus (%) (*n* = 313)	19/213 (8.9%)	11/100 (11.0%)	0.706		
Stroke (%) (*n* = 313)	5/213 (2.3%)	5/100 (5.0%)	0.299		
WFNS Grade, median (IQR)	II (I–II)	IV (IV–V)	<0.001 *^†^		
I (%)	102 (44.0%)	9 (8.5%)			
II (%)	74 (31.9%)	11 (10.4%)			
III (%)	11 (4.7%)	6 (5.7%)			
IV (%)	20 (8.6%)	31 (29.2%)			
V (%)	25 (10.8%)	49 (46.2%)	<0.001 *^‡^	5.27 (2.48–11.19)	<0.001 *
Aneurysm size (mm), mean (SD) (*n* = 318)	5.5 (2.6) (*n* = 223)	6.9 (3.6) (*n* = 95)	<0.001 *^†^	1.03 (0.92–1.16)	0.665
Posterior circulation (VA, BA, PCA, PICA) (%)	18 (7.8%)	10 (9.4%)	0.760		
Fisher CT group	3.0 (3.0–3.0)	3.0 (3.0–3.0)	<0.001 *^†^		
1–2 (%)	30 (12.9%)	8 (7.5%)		1.07 (0.37–3.13)	0.895
3 (%)	189 (81.5%)	72 (67.9%)		1.00 (Reference)	
4 (%)	13 (5.6%)	26 (24.5%)	<0.001 *^§^	12.06 (4.18–34.83)	<0.001 *
Surgical procedure					
Endovascular coiling, not surgical clipping (%endovascular coiling)	122 (52.6%)	44 (41.5%)	0.076		
Spinal drainage (%)	103 (44.4%)	45 (42.5%)	0.829		
Ventricular drainage (%)	57 (24.6%)	52 (49.1%)	<0.001 *	2.27 (1.04–4.98)	0.041 *
Cisternal drainage (%)	39 (16.8%)	32 (30.2%)	0.008 *	2.04 (0.82–5.09)	0.125
Cerebral vasospasm prophylaxis and other medications					
Fasudil (%)	67 (28.9%)	38 (35.8%)	0.247		
Cilostazol (%)	161 (69.4%)	74 (69.8%)	1.000		
Statin (%)	89 (38.4%)	56 (52.8%)	0.018 *	3.24 (1.48–7.08)	0.003 *
Nicardipine (%)	2 (0.9%)	4 (3.8%)	0.080		
Antiepileptic drug (%)	86 (37.1%)	36 (34.0%)	0.667		
Clazosentan discontinuation (%)	5 (2.2%)	24 (22.6%)	<0.001 *	5.12 (1.41–18.60)	0.013 *
Outcomes and complications					
Angiographic vasospasm (%) (*n* = 324)	26/225 (11.6%)	34/99 (34.3%)	<0.001 *		
Symptomatic vasospasm (%) (*n* = 319)	7/225 (3.1%)	18/94 (19.1%)	<0.001 *	10.58 (3.28–34.16)	<0.001 *
IVR against cerebral vasospasm (%)	4 (1.7%)	6 (5.7%)	0.077		
Cerebral infarction (%) (*n* = 323)	27/225 (12.0%)	33/98 (33.7%)	<0.001 *		
Cerebral complication (%)	44 (19.0%)	52 (49.1%)	<0.001 *		
Systemic complication (%)	36 (15.5%)	44 (41.5%)	<0.001 *	7.62 (3.28–17.74)	<0.001 *

Five imputed datasets were generated, and pooled estimates are presented. Variables significant in the univariable analysis were included in the multivariable model, along with age, sex, WFNS Grade, and Fisher CT group. These analyses represent non-causal associations. Given the lack of time-resolved data on treatment discontinuation, the results should be interpreted as descriptive and exploratory. Abbreviations: BA, basilar artery; CI, confidence interval; CT, computed tomography; Fasudil, fasudil hydrochloride hydrate; IVR, interventional radiology; IQR, interquartile range; mRS, modified Rankin Scale; OR, odds ratio; PCA, posterior cerebral artery; PICA, posterior inferior cerebellar artery; SD, standard deviation; VA, vertebral artery; WFNS, World Federation of Neurosurgical Societies; *, *p* < 0.05. ^†^, treated as an ordinal variable, and tested by the Mann–Whitney U test. ^‡^, treated as a binomial variable, I–IV or V, and tested by the chi-square test. ^§^, treated as a three-categorical variable—1 and 2, 3, or 4—and tested by the chi-square test.

**Table 7:** Previous reports on adverse events and discontinuation of clazosentan.

Study	Author, Year	Number of Patients	Age, Mean (SD)	WFNS Grade	Fisher CT Group or Clot Distribution	Concomitant Prophylaxis	Adverse Event Proportion	Discontinuation Proportion Due to Adverse Event
CONSCIOUS-1	Macdonald et al., 2008 [10]	313 (107, 1 mg/h; 110, 5 mg/h; 96, 15 mg/h)	51 (10); 51 (11); 51 (11)	I 43%, II 33%, III–V 24% (1 mg/h); I 41%, II 32%, III–V 27% (5 mg/h); I 46%, II 29%, III–V 25% (15 mg/h)	Diffuse (≥20 mm) or localized (<20 mm) thick (≥4 mm) subarachnoid clot	Oral nimodipine (proportion not described)	Pulmonary complication 44%, hypotension 6%, anemia 25% (1 mg/h); Pulmonary complication 44%, hypotension 12%, anemia 29% (5 mg/h); Pulmonary complication 39%, hypotension 12%, anemia 20% (15 mg/h)	1% due to hypotension
CONSCIOUS-2 (clipping)	Macdonald et al., 2011 [8]	764 (5 mg/h)	52.0 (10.9)	I 51%, II 26%, III 5%, IV 16%, V 1%	Diffuse (≥20 mm) or localized (<20 mm) thick (≥4 mm) subarachnoid clot	Oral nimodipine (≥90%)	Pulmonary complication 34%, hypotension 12%, hepatobiliary event 20%, anemia 22%	8.5% (5 mg/h)
CONSCIOUS-3 (coiling)	Macdonald et al., 2012 [9]	382 (194, 5 mg/h; 188, 15 mg/h)	52 (11); 53.6 (11)	I 51%, II 25%, III 3%, IV 19%, V 2% (5 mg/h); I 55%, II 28%, III 2%, IV 14%, V 1% (15 mg/h)	Thick (short axis ≥ 4 mm) subarachnoid clot	Oral nimodipine (≥90%)	Pulmonary complication 36%, hypotension 11%, hepatobiliary event 13%, anemia 13% (5 mg/h); Pulmonary complication 37%, hypotension 16%, hepatobiliary event 15%, anemia 13% (15 mg/h)	8.8% (5 mg/h); 11.7% (15 mg/h)
Japan phase 3 (coiling + clipping)	Endo et al., 2022 [12]	218 (109 clipping, 109 coiling; 10 mg/h)	59.1 (17.1)	I 39.6%, II 34.7%, III 3.7%, IV 22.0%, V 0%	Group 3 100%	None	Total 35.3% (Pulmonary edema 11.9%, pleural effusion 16.5%, cerebral edema 6.0%, anemia 16.5%)	7.8%
REACT	Mayer et al., 2025 [11]	202 (15 mg/h)	53.7 (10.4)	I 52.0%, II 27.7%, III 4.0%, IV 8.9%, V 7.4%	Thick and diffuse clot (>4 mm, ≥3 cisterns)	Oral or intravenous nimodipine (95.5%)	Pulmonary complication 25.6%, fluid retention 19.8%, hypotension 10.1%, cerebral edema 13.0%	5.8%

Abbreviations: CT, computed tomography; SD, standard deviation; WFNS, World Federation of Neurosurgical Societies.

**Table 8:** Real-world observational studies on adverse events and discontinuation of clazosentan.

Author, Year	Number of Patients	Age	WFNS Grade	Fisher CT Group	Concomitant Prophylaxis	Adverse Event Proportion	Factors Associated with Adverse Events	Discontinuation Proportion	Factors Associated with Discontinuation
Muraoka et al., 2023 [20]	47	Mean 64.4 (SD 15.0)	I 23.4%, II 29.8%, III 19.1%, IV 12.8%, V 14.9%	1 4.3%, 2 17.0%, 3 59.6%, 4 19.1%	Fasudil 38.3%, cilostazol 78.7%, ozagrel 31.9%, diuretic 42.6%	Pulmonary edema 40.4%, hypotension 34.0%	Pulmonary edema: higher age, higher fluid balance, minimum albumin; hypotension: not significant.	17.0%	Smoking (univariable analysis)
Maeda et al., 2024 [18]	18	Median 74 [IQR 54.5–85.5]	I–III 66.7%, IV–V 33.3%	3 83.3%	Fasudil 100%	Pulmonary edema 11.1%, pleural effusion 27.8%, fluid retention 38.9%	Higher age (univariable analysis)	0%	Not performed
Sakata et al., 2024 [25]	81	63 [51–76]	I 56.8%, II 17.3%, III 3.7%, IV 16.1%, V 6.2%	1 0%, 2 6.2%, 3 91.4%, 4 2.5%	None	Pulmonary edema 19.8%	Higher age	2.5% (1 fluid retention, 1 hypotension)	Not performed
Mochizuki et al., 2024 [19]	32	59.5 [54.8–75.5]	I 9.4%, II 12.5%, III 6.3%, IV 21.9%, V 50.0%	Not described	Cilostazol 25%, statin 25%, eicosapentaenoic acid 23%, ozagrel 3.1%	Pleural effusion 56.2%, cerebral edema 12.5%	Not performed	3.1%	Not performed
Mutoh et al., 2024 [22]	6 (Age ≥ 75 years)	80.3 (5.2)	I 33.3%, II 16.7%, III 16.7%, IV 0%, V 33.3%	Not described	None	Pleural effusion 50%, hypoxia 50%, hypotension 33%, heart failure 33%, pulmonary edema 17%	Not performed	50%	Low urinary volume, increased body weight after surgery (univariable analysis)
Mutoh et al., 2024 [23]	40	Clazosentan completion 60.5 (12.6); discontinuation 67.7 (14.0)	Mean ± SD: Clazosentan completion 2.2 ± 1.5; discontinuation 2.2 ± 1.7	Not described	None	22% (Pleural effusion, hypoxia, hypotension)	Not performed	22.5%	Day-to-day urine volume variation, Age ≥ 76 years
Kajiwara et al., 2025 [16]	27	58.8 (14.1)	I 25.9%, II 37.0%, III 18.5%, IV 18.5%, V 0%	1 0%, 2 11.1%, 3 70.4%, 4 18.5%	Cilostazol 3.9%	Pleural effusion 48.2%	Not performed	3.7% (pulmonary edema)	Not performed
Okumura et al., 2025 [24]	43	60 [21–89]	I 42%, II 30%, III 2%, IV 19%, V 7%	1 0%, 2 14%, 3 58%, 4 28%	Cilostazol 93%, statin 88%	Pulmonary edema 34.9%	Not performed	Not included	Not performed
Akamatsu et al., 2025 [14]	69	60.9 (16.5)	I 3%, II 30%, III 16%, IV 17%, V 3%	0–2 21.8%, 3–4 78.2%	Goreisan (Japanese herbal kampo) 60.0%, nicardipine 100%	Pulmonary edema 5.8%, hyponatremia 4.3%	Hyponatremia: absence of goreisan (univariable analysis)	5.8% (pulmonary edema)	Not performed
Kondo et al., 2025 [17]	166	Mean 66.1	I 22.9%, II 23.5%, III 10.8%, IV 14.5%, V 22.7%	1 1.8%, 2 6.6%, 3 68.7%, 4 22.9%	Cilostazol 47.6%, statin 42.2%	15.7%	Direct surgery, absence of strict protocol (routine thoracic CT, monotherapy of clazosentan, infusion volume with 1 mL/kg/h, daily fluid balance 0 to +500 mL maintaining normovolemia)	5.4%	Not performed
Ando-Matsuoka et al., 2025 [15]	241 (Adverse Drug Event Report-based study)	75 [62.5–75]	Not described	Not described	Fasudil 36.9%, cilostazol 35.3%, ozagrel 18.3%, nicardipine 11.2%	Fluid retention 47.3%	Age ≥ 71 years, concomitant Fasudil use	Not described	Not performed
Muraoka et al. and Takeuchi et al., 2025 [21,29]	161	61.8 (12.9)	I 20.5%, II 34.2%, III 16.8%, IV 14.9%, V 13.7%	1 3.7%, 2 19.9%, 3 55.9%, 4 20.5%	Cilostazol 58.4%, statin 53.4%, ozagrel 8.7%	Pulmonary complication 5.6%, hypotension 1.9%, cerebral edema 6.8%	Not performed.	14.3% (13 cardiopulmonary complications, 7 cerebral edema, 3 hypotension)	Not performed
Sugiyama et al., 2025 [28]	80	59.1 (13.0)	I 37.5%, II 21.3%, III 2.5%, IV 25.0%, V 13.8%	1 0%, 2 21.3%, 3 62.5%, 4 16.3%	Cilostazol 89.8%	Pulmonary edema 8.8%, pleural effusion 12.5%, cerebral edema 5%, hypotension 5%, anemia 8%	Age ≥ 76 years, low albumin, total protein, hematocrit levels, large cardiothoracic ratio at admission (univariable analysis)	3.8% (pulmonary complication, hypotension, systemic inflammation)	Not performed
Sekimoto et al., 2025 [27]	32	57.9 (17.1)	I–III 50%, IV–V 50%	1 3.1%, 2 3.1%, 3 84.4%, 4 9.4%	Fasudil 6.3%, cilostazol 43.8%, statin 31.3%	Fluid retention 46.8%	Higher age, daily fluid balance before day 3	12.5% (1 pulmonary edema, 3 systemic complications)	Not performed
Sakata et al., 2026 [26]	187	66 [51–76]	I 37.4%, II 25.7%, III 9.6%, IV 15.0%, V 12.3%	1 0%, 2 5.4%, 3 87.7%, 4 7.0%	None	Pulmonary edema 22.5%	Not performed.	5.9% (8 fluid retention, 3 hypotension)	Not performed
Ours, 2026	372	64.2 (14.3)	I 31.2%, II 25.5%, III 5.1%, IV 15.3%, V 22.8%	1 0.3%, 2 10.8%, 3 75.5%, 4 13.4%	Fasudil 31.5%, cilostazol 70.4%, statin 42.7%, nicardipine 2.4%	Not systematically assessed	Not assessed.	8.3% (pulmonary complication 29.0%, cardiac complications 25.8%, hypotension 12.9%, cerebral edema 12.9%, deterioration of general condition 9.7%, intracranial hemorrhage 6.5%, septic shock 3.2%)	Age ≥ 76 years, WFNS Grade V, larger aneurysm size, Fisher CT group 4, and Fasudil use

Studies are presented for descriptive contextual comparison and do not represent a systematic review. Abbreviations: CT, computed tomography; Fasudil, fasudil hydrochloride hydrate; IQR, interquartile range; SD, standard deviation; WFNS, World Federation of Neurosurgical Societies.

## Data Availability

De-identified data are available from the corresponding author upon reasonable request in accordance with the policy of the Shimane Prefectural Central Hospital Review Board.

## Supplementary Materials

The following supporting information can be downloaded at https://www.mdpi.com/article/10.3390/jcm15176744/s1: Supplementary File S1. Supplementary Method S1: Details about the study protocol of DCI-Japan; Supplementary File S2. Supplementary Method S2: Detailed methods; Supplementary Table S1: Multivariable Firth’s penalized logistic regression model identifying clinical variables associated with clazosentan discontinuation without multiple imputations (*n* = 352). Supplementary Table S2: Secondary exploratory multivariable Firth’s penalized logistic regression model examining clinical variables correlated with poor discharge outcomes (mRS 3–6) without multiple imputations (*n* = 309). Supplementary Table S3: Multivariable Firth’s penalized logistic regression model identifying clinical variables associated with poor outcomes (mRS 3–6) at 6 months without multiple imputations (*n* = 278). Supplementary Table S4: Clinical characteristics of patients aged ≥75 years with aneurysmal SAH treated with clazosentan (*n* = 111). Supplementary Table S5: Outcomes of patients aged ≥75 years with aneurysmal SAH treated with clazosentan (*n* = 111). STROBE Checklist: STROBE Statement—Checklist of items that should be included in reports of observational studies.

## Abbreviations

The following abbreviations are used in this manuscript:

ACA	anterior cerebral artery
ACoA	anterior communicating artery
aSAH	aneurysmal subarachnoid hemorrhage
AVS	angiographic vasospasm
BA	basilar artery
CI	confidence interval
CT	computed tomography
DCI	delayed cerebral ischemia
DCI Japan	Database of Cohort Study for Outcome of SAH In Japan
Fasudil	fasudil hydrochloride hydrate
ICA	internal carotid artery
IVR	interventional radiology
IQR	interquartile range
MCA	middle cerebral artery
mRS	modified Rankin Scale
OR	odds ratio
PCA	posterior cerebral artery
PICA	posterior inferior cerebellar artery
RCT	randomized controlled trial
Q1–Q3	25th–75th percentile
SAH	subarachnoid hemorrhage
SD	standard deviation
SVS	symptomatic vasospasm
VA	vertebral artery
VS	vasospasm
WFNS	World Federation of Neurosurgical Societies
