# Supplementary material for: The Clinical Factors Associated with Clazosentan Discontinuation and Exploratory Associations with Discharge and Six-Month Outcomes in Aneurysmal Subarachnoid Hemorrhage: A Multicenter DCI Japan Registry Study
Source: J Clin Med. 2026 Aug 30;15(17):6744. doi: 10.3390/jcm15176744 (PMC13566513; doi:10.3390/jcm15176744)
Supplement: Supplementary file 1 [file jcm-15-06744-s001.zip › 04JCM Sup Tables REV1 TC.pdf]

Supplementary Table S1

Multivariable Firth's penalized logistic regression model identifying clinical variables associated with clazosentan discontinuation without multiple imputation (n=352)

Variable	OR (95% CI)	p value
Age ≥76 years	2.40 (1.04–5.58)	0.039*
Female sex	1.12 (0.48–2.56)	0.786
WFNS Grade V (vs. I–IV)	6.89 (2.74–17.28)	<0.001*
Aneurysm size (mm)	1.14 (1.01–1.27)	0.021*
Fisher CT group		
1–2	0.42 (0.12–1.34)	0.137
3	1.00 (Reference)	
4	2.49 (1.02–6.10)	0.044*
Fasudil use	3.81 (1.67–8.71)	0.002*
Nicardipine use	1.20 (0.50–2.80)	0.687

Aneurysm size was missing in 20 patients; these cases were excluded from the complete-case model (n=352). Variables that were significant in the univariable analysis (Table 1) were included in the multivariable model, along with age, sex, WFNS Grade, and Fisher CT group.

Abbreviations: CI, confidence interval; CT, computed tomography; Fasudil, fasudil hydrochloride hydrate; OR, odds ratio; WFNS, World Federation of Neurosurgical Societies; *, $p < 0.05$.

Supplementary Table S2. Secondary exploratory multivariable Firth's penalized logistic regression model examining clinical variables correlated with poor discharge outcomes (mRS 3–6) without multiple imputations (n = 309).

Variable	OR (95% CI)	p value
Age ≥76 years	7.36 (3.44–15.70)	<0.001*
Female sex	1.21 (0.62–2.36)	0.588
Stroke	2.59 (0.42–16.10)	0.303
WFNS Grade V	5.06 (2.35–10.91)	<0.001*
Aneurysm size (mm)	1.07 (0.96–1.20)	0.235
Fisher CT group 1–2	0.62 (0.20–1.93)	0.381
Fisher CT group 3	1.00 (Reference)	–
Fisher CT group 4	10.07 (3.10–32.70)	<0.001*
Ventricular drainage	2.04 (1.08–3.85)	0.028*
Statin use	3.24 (1.64–6.39)	<0.001*
Clazosentan discontinuation	7.16 (1.33–38.50)	0.022*
Symptomatic vasospasm	3.08 (1.02–9.30)	0.047*
Systemic complication	3.73 (1.63–8.55)	0.002*

At least one missing value among the variables included in the multivariable model was present in 63 patients; therefore, 309 patients were included in the complete-case analysis. Variables significant in the univariable analysis (**Table 5**) were included in the multivariable model, along with age, sex, WFNS Grade, and Fisher CT group. These analyses represent non-causal associations. Given the lack of time-resolved data on treatment discontinuation, the results should be interpreted as descriptive and exploratory.

Abbreviations: CI, confidence interval; CT, computed tomography; mRS, modified Rankin Scale; OR, odds ratio; SVS, symptomatic vasospasm; WFNS, World Federation of Neurosurgical Societies; *, $p < 0.05$.

Supplementary Table S3

Multivariable Firth's penalized logistic regression model identifying clinical variables associated with poor outcomes (mRS 3–6) at 6 months without multiple imputation (n=278)

Variable	OR (95% CI)	p value
Age ≥76 years	4.73 (2.09–10.69)	<0.001*
Female sex	1.08 (0.50–2.34)	0.849
Stroke	0.72 (0.07–7.25)	0.784
WFNS Grade V	4.71 (2.08–10.66)	<0.001*
Aneurysm size (mm)	1.04 (0.92–1.18)	0.546
Fisher CT group 1–2	1.01 (0.29–3.50)	0.985
Fisher CT group 3	1.00 (Reference)	
Fisher CT group 4	10.61 (3.38–33.32)	<0.001*
Ventricular drainage	1.76 (0.71–4.33)	0.219
Cisternal drainage	2.45 (0.89–6.77)	0.083
Statin use	3.59 (1.55–8.33)	0.003*
Clazosentan discontinuation	3.83 (0.90–16.33)	0.070
Symptomatic vasospasm	9.14 (2.71–30.85)	<0.001*
Systemic complication	4.81 (1.87–12.37)	0.001*

Among 338 patients with available 6-month mRS, missing values were present for past history of stroke (n = 25), aneurysm size (n = 20), and symptomatic vasospasm (n = 19). After excluding patients with at least one missing value among the variables included in the multivariable model, 278 patients were included in the complete-case analysis. Variables that were significant in the univariable analysis (**Table 6**) were included in the multivariable model, along with age, sex, WFNS Grade, and Fisher CT group.

Abbreviations: CI, confidence interval; CT, computed tomography; mRS, modified Rankin Scale; OR, odds ratio; WFNS, World Federation of Neurosurgical Societies; *, $p < 0.05$.

Supplementary Table S4

Clinical characteristics of patients aged ≥75 years with aneurysmal SAH treated with clazosentan (n=111)

Variable	Overall (n=111)	Completion group (n=91, 82.0%)	Discontinuation group (n=20, 18.0%)	p value
Age (years), mean (SD)†	80.5 (4.8)	80.6 (4.9)	79.8 (4.7)	0.393
Female sex (%)	91 (82.0%)	76 (83.5%)	15 (75.0%)	0.353
Past history				
Hypertension (%)	49/100 (49.0%)	41/83 (49.4%)	8/17 (47.1%)	1.000
Diabetes mellitus (%)	10/100 (10.0%)	7/83 (8.4%)	3/17 (17.6%)	0.367
Stroke (%)	10/100 (10.0%)	10/83 (12.0%)	0/17 (0.0%)	0.204
WFNS Grade, median (IQR)	3 (2–5)	2 (1–5)	4 (4–5)	0.006*†
1 (%)	26 (23.4%)	25 (27.5%)	1 (5.0%)	
2 (%)	25 (22.5%)	24 (26.4%)	1 (5.0%)	
3 (%)	6 (5.4%)	4 (4.4%)	2 (10.0%)	
4 (%)	21 (18.9%)	13 (14.3%)	8 (40.0%)	
5 (%)	33 (29.7%)	25 (27.5%)	8 (40.0%)	0.401‡
Aneurysm size (mm), mean (SD)	6.5 (3.8) (n=107)	6.2 (3.5) (n=88)	8.1 (4.9) (n=19)	0.066†
Aneurysm location				
ACA or ACoA (%)	42 (37.8%)	37 (40.7%)	5 (25.0%)	
ICA (%)	38 (34.2%)	31 (34.1%)	7 (35.0%)	
MCA (%)	19 (17.1%)	13 (14.3%)	6 (30.0%)	
VA, BA, PCA, PICA (%)	12 (10.8%)	10 (11.0%)	2 (10.0%)	1.000¶
Fisher CT group, median (IQR)	3 (3–3)	3 (3–3)	3 (3–4)	0.018*†
1–2 (%)	11 (9.9%)	9 (9.9%)	2 (10.0%)	
3 (%)	82 (73.9%)	72 (79.1%)	10 (50.0%)	
4 (%)	18 (16.2%)	10 (11.0%)	8 (40.0%)	0.006*§
Surgical procedure				
Endovascular coiling, not surgical clipping (%)	62 (55.9%)	51 (56.0%)	11 (55.0%)	1.000
Spinal drainage (%)	47 (42.3%)	38 (41.8%)	9 (45.0%)	0.987
Ventricular drainage (%)	36 (32.4%)	30 (33.0%)	6 (30.0%)	1.000
Cisternal drainage (%)	16 (14.4%)	16 (17.6%)	0 (0.0%)	0.071
Cerebral vasospasm prophylaxis and other medications				
Fasudil (%)	38 (34.2%)	28 (30.8%)	10 (50.0%)	0.167
Cilostazol (%)	73 (65.8%)	59 (64.8%)	14 (70.0%)	0.857

Statin (%)	49 (44.1%)	42 (46.2%)	7 (35.0%)	0.509
Nicardipine (%)	3 (2.7%)	1 (1.1%)	2 (10.0%)	0.083
Antiepileptic drug (%)	43 (38.7%)	37 (40.7%)	6 (30.0%)	0.527

Abbreviations: ACA, anterior cerebral artery; ACoA, anterior communicating artery; BA, basilar artery; CT, computed tomography; Fasudil, fasudil hydrochloride hydrate; ICA, internal carotid artery; IQR, interquartile range; MCA, middle cerebral artery; PCA, posterior cerebral artery; PICA, posterior inferior cerebellar artery; SAH, subarachnoid hemorrhage; SD, standard deviation; VA, vertebral artery; WFNS, World Federation of Neurosurgical Societies; *, $p < 0.05$.

†, tested using the Mann–Whitney U test.

‡, treated as a binomial variable, I–IV or V, and tested by the chi-square test.

§, treated as a three-categorical variable: 1 and 2, 3, or 4, and tested by the chi-square test.

¶, treated as a binomial variable, anterior circulation or posterior circulation, and tested by the chi-square test.

Supplementary Table S5

Outcomes of patients aged ≥75 years with aneurysmal SAH treated with clazosentan (n=111)

	Overall (n=111)	Completion group (n=91, 82.0%)	Discontinuation group (n=20, 18.0%)	p value
mRS score at discharge				<0.001*†
0 (%)	8 (7.2%)	8 (8.8%)	0 (0.0%)	
1 (%)	10 (9.0%)	10 (11.0%)	0 (0.0%)	
2 (%)	11 (9.9%)	11 (12.1%)	0 (0.0%)	
3 (%)	18 (16.2%)	16 (17.6%)	2 (10.0%)	
4 (%)	18 (16.2%)	14 (15.4%)	4 (20.0%)	
5 (%)	34 (30.6%)	27 (29.7%)	7 (35.0%)	
6 (%)	12 (10.8%)	5 (5.5%)	7 (35.0%)	
Poor outcome at discharge, mRS 3–6 (%)	82/111 (73.9%)	62/91 (68.1%)	20/20 (100.0%)	0.008*
mRS score at 6 months (n=93)				0.006*†
0 (%)	17/93 (18.3%)	17/75 (22.7%)	0/18 (0.0%)	
1 (%)	17/93 (18.3%)	15/75 (20.0%)	2/18 (11.1%)	
2 (%)	9/93 (9.7%)	9/75 (12.0%)	0/18 (0.0%)	
3 (%)	3/93 (3.2%)	1/75 (1.3%)	2/18 (11.1%)	
4 (%)	10/93 (10.8%)	7/75 (9.3%)	3/18 (16.7%)	
5 (%)	24/93 (25.8%)	17/75 (22.7%)	7/18 (38.9%)	
6 (%)	13/93 (14.0%)	9/75 (12.0%)	4/18 (22.2%)	
Poor outcome at 6 months, mRS 3–6 (%)	50/93 (53.8%)	34/75 (45.3%)	16/18 (88.9%)	0.002*
Complications				
Angiographic vasospasm (%)	21/105 (20.0%)	16/89 (18.0%)	5/16 (31.2%)	0.305
Symptomatic vasospasm (%)	11/105 (10.5%)	6/88 (6.8%)	5/17 (29.4%)	0.016*
IVR against cerebral vasospasm (%)	4 (3.6%)	3 (3.3%)	1 (5.0%)	0.554
Cerebral infarction (%)	25/106 (23.6%)	18/89 (20.2%)	7/17 (41.2%)	0.114
Cerebral complication (%)	35 (31.5%)	24 (26.4%)	11 (55.0%)	0.026*
Systemic complication (%)	36 (32.4%)	25 (27.5%)	11 (55.0%)	0.034*

Abbreviations: IVR, interventional radiology; mRS, modified Rankin Scale; SAH, subarachnoid hemorrhage.

*, $p < 0.05$.

†, treated as an ordinal variable, and tested by the Mann-Whitney U test.
