# Supplementary material for: The Clinical Factors Associated with Clazosentan Discontinuation and Exploratory Associations with Discharge and Six-Month Outcomes in Aneurysmal Subarachnoid Hemorrhage: A Multicenter DCI Japan Registry Study
Source: J Clin Med. 2026 Aug 30;15(17):6744. doi: 10.3390/jcm15176744 (PMC13566513; doi:10.3390/jcm15176744)
Supplement: Supplementary file 1 [file jcm-15-06744-s001.zip › 05 SupFileS2 JCM METHODS REV1 TC.pdf]

Supplementary File S2 SUPPLEMENTARY METHODS

2.3 Consistent treatment protocol of general management for aneurysmal SAH

Treatment protocol for aneurysmal SAH (aSAH) across all institutions followed the Japanese Guidelines for the Management of Stroke 2021.[30] Preoperative management included nicardipine to maintain normovolemia and a systolic blood pressure <130 mmHg. Surgical indications followed the Japanese Guidelines for the Management of Stroke 2021: patients with WFNS Grades I–III were eligible for aneurysm treatment, while those with Grades IV and V were generally excluded unless they were young, middle-aged, or had a significant hematoma or hydrocephalus. Definitive treatment (surgical clipping or endovascular coiling) was performed less than 4 days of onset by certified neurosurgeons following a thorough discussion. Postoperative care included prevention of VS, rehabilitation, and management of systemic complications. Symptomatic VS (SVS) was treated with intra-arterial Fasudil or percutaneous transluminal angioplasty as needed.

2.4 Inclusion and exclusion criteria

A flow diagram of patient selection is shown in **Figure 1**. This study included patients with aSAH who underwent surgical clipping or endovascular coil embolization for a ruptured aneurysm less than 4 days of symptom onset. Exclusion criteria were: failure to identify a ruptured aneurysm on imaging; non-saccular aneurysms (e.g., dissecting, fusiform, infectious, or traumatic); prehospital modified Rankin Scale (mRS) ≥2 or unavailable; absence of definitive treatment; treatments other than simple surgical clipping or coiling (e.g., wrapping, trap and bypass, parent artery occlusion, decompression, or stent-assisted coiling); complex or crossover treatments; missing or inaccurate onset or treatment dates; or treatment initiated ≥4 days after onset. Among patients meeting these criteria, those who received clazosentan after definitive treatment were further included in the final analysis.

2.5 Definition of VS

Cerebral VS was assessed using CT angiography or digital subtraction angiography according to local protocols, typically immediately after surgery, on postoperative days 1, 7, and 14, and when new neurological deficits emerged. The classification into angiographic VS (AVS), SVS, and cerebral infarction followed the conventional model of vasoconstriction leading to ischemia.[31]

AVS was defined as ≥50% narrowing in any cerebral artery on angiography compared with preoperative baseline, excluding changes caused by atherosclerosis, catheter-induced spasm, or vessel hypoplasia. Diagnosis was based solely on imaging.[31]

SVS was defined as neurological deficits occurring during the VS phase (typically days 3–14 after aSAH onset); the appearance of focal neurological signs, or a ≥2-point decrease in Glasgow Coma Scale sustained for at least 1 hour, or both, was considered indicative of ischemia attributable to VS after excluding other potential causes of worsening (e.g., hydrocephalus, seizures, metabolic derangement, infection, or oversedation).[32]

Cerebral infarction was defined as new infarcts occurring within 6 weeks of SAH on CT, magnetic resonance imaging, or autopsy, which were absent on early postoperative imaging and not attributable to surgery, catheters, or hematoma. Infarcts from non-VS mechanisms (e.g., cardioembolism, atherothrombosis) were also included.[32] In the present study, SVS was selected as the primary VS-related outcome among VS-related variables because it represents a clinically apparent ischemic event attributable to VS and could be consistently identified across participating centers, whereas DCI encompasses heterogeneous mechanisms beyond SVS.

2.8 Statistical analyses

Normality of continuous variables was assessed using the Shapiro–Wilk test. Continuous variables with normal distribution were described as means (standard deviation [SD]) and compared using a t-test. Continuous variables that were not normally distributed and ordinal variables were described as medians [25th–75th percentile; Q1–Q3] and compared using the Mann-Whitney U test. Categorical variables were described as actual numbers and percentages, and the proportions were compared using chi-square or Fisher's exact tests. Significant variables from univariable analyses were included in the multivariable analysis, along with age (≥76 years), sex, WFNS Grade (Grade V vs I–IV), and Fisher CT group (1–2, 3, or 4). These categorical variables were included to evaluate the influence of underrepresented patient subgroups in the Japanese phase 3 RCT.[10] Given the exploratory nature of this real-world observational study and the lack of prior data on clazosentan discontinuation, no statistical power calculation was conducted before the study, and the sample size was based on available data.

A multivariable logistic regression model was used to investigate the association between each outcome and the factors described above. To prevent overfitting, given the limited number of events, the number of covariates included in each multivariable model was strictly based on clinical relevance and event frequency. If there were variables with missing values, we performed five imputations, and the pooled results were presented as the main results; case-wise analyses were shown in the **Supplementary Tables S1–3**. Because of an imbalance in outcome frequencies, Firth's penalized likelihood method was applied. If there were variables with a correlation coefficient of 0.8 or higher and suggested high multicollinearity, one was selected for use based on clinical reasonability. Odds ratios (ORs) and 95% confidence intervals (CIs) of items were determined. Statistical significance was set at a two-tailed $p < 0.05$. SPSS Statistics version 31.0.0 (IBM Corp., Armonk, NY, USA), Python 3.9.0, Statsmodels 0.14.6, Pandas 2.0.2, and Matplotlib 3.5.1 were used.
